# Mucocutaneous leishmaniasis in a cocaine user: diagnostic and therapeutic knowledge

**DOI:** 10.1590/0037-8682-0040-2020

**Published:** 2020-06-22

**Authors:** Lissiê Lunardi Sbroglio, Viviane Maria Maiolini, Irene Machado Moraes Alvarenga Rabelo, Gabriela Almeida Giraldelli, Luciana Patrícia Tuccori, Rodrigo Guimarães Cunha

**Affiliations:** 1 Universidade do Estado do Rio de Janeiro, Programa de Residência Médica em Dermatologia, Rio de Janeiro, RJ, Brasil.; 2 Universidade do Estado do Rio de Janeiro, Programa de Pós-Graduação em Dermatologia, Rio de Janeiro, RJ, Brasil.; 3 Universidade do Estado do Rio de Janeiro, Programa de Residência Médica em Infectologia, Rio de Janeiro, RJ, Brasil.; 4 Universidade do Estado do Rio de Janeiro, Departamento de Infectologia, Rio de Janeiro, RJ, Brasil.

**Keywords:** Mucocutaneous Leishmaniasis, Cocaine related disorders, Infectious Disease Medicine

## Abstract

Mucocutaneous leishmaniasis (MCL) is a chronic infection that can affect the skin and mucous membranes. We report a case of oral, nasopharyngeal, and penile lesions in a 35-year-old cocaine user. The patient presented with ulcerated lesions in 2014. Histopathologic analysis revealed amastigotes, and serological test results were positive for leishmaniasis. Systemic therapy with meglumine antimoniate was administered; however, the patient failed to present for follow-up. In 2018, he returned with nasal collapse, and another histopathologic test confirmed MCL. This case illustrates the importance of careful differential diagnosis of skin and mucous ulcers to identify the particular pathology.

## INTRODUCTION

Leishmaniasis is a chronic infection caused by several protozoan species of the genus *Leishmania*. The infection is transmitted by sandflies, and a wide variety of domestic and wild vertebrates serve as parasitic reservoirs; however, humans are accidental hosts in the Americas. Leishmaniasis is endemic to the Middle East, North Africa, parts of Europe, and Central and South America^1^ ^,^ ^2^.

There are four major clinical forms of leishmaniasis: cutaneous leishmaniasis (CL), mucocutaneous leishmaniasis (MCL), diffuse cutaneous leishmaniasis (DCL), and visceral leishmaniasis^1^ ^,^ ^3^. The infecting species and the interaction with the host will determine how the infection presents. 

In CL, an elevated erythematous papule develops at the bite site weeks or even years later. This papule leads to an ulcer with raised edges, regular contours, and coarse granulation, either covered or not covered with seropurulent exudate. MCL can affect the skin and mucosa. Mucosal involvement occurs most commonly with species belonging to the subgenus *Leishmania viannia* and may affect the nasal passage, pharynx, larynx, or genitalia, leading to extensive tissue destruction. The infection spread results from the metastatization of the skin lesion by contiguity or through the lymphatic pathway and may occur concurrently with or years after healing of the primary skin lesion. DCL is characterized by progressive chronic evolution and usually affects young children. In Brazil, the etiologic agent responsible for DCL is *Leishmania (Leishmania) amazonensis*. In endemic areas, asymptomatic individuals can present with positive delayed hypersensitivity skin testing^3^.

## CASE REPORT

**FIGURE 1:**
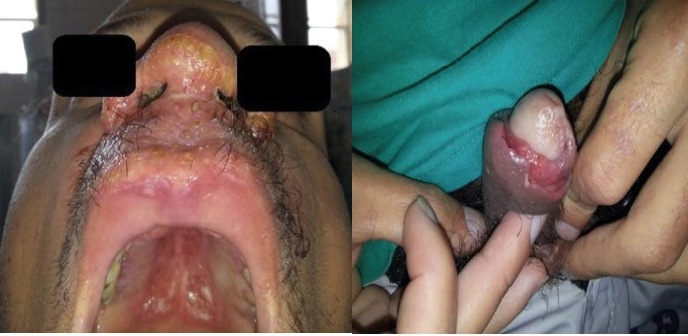
Characteristic ulcerated lesion with exudation and infiltration in the nasal base, filter, palate, and balanoprepucial sulcus.

**FIGURE 2:**
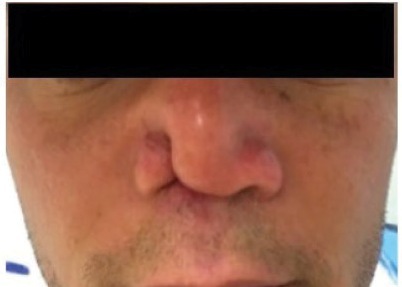
Nasal collapse on the right.

In 2014, a 35-year-old male resident of Rio de Janeiro was admitted with ulcerated lesions on his nasal septum, nasal cavity, palate, and balanopreputial sulcus (Figure 1). The patient had a past history of alcohol and cocaine abuse. The lesions initially appeared in 2013 as erythematous and painful papules that grew progressively larger. Additional symptoms included headache, nasal obstruction, asthenia, and weight loss. The initial differential diagnoses included MCL, paracoccidioidomycosis, neoplasia, granulomatosis with polyangiitis, and tuberculosis. The diagnosis of MCL was confirmed on the basis of positive IgG and IgM serology and histopathological examinations of the nasal mucosa and larynx. Nasal mucosa histopathological analysis showed dermal infiltration of histocytes, lymphocytes, and plasma cells in addition to some dilated vessels. The histopathologic analysis of the larynx showed extensive ulceration and inflammatory infiltrates, and a single macrophage with amastigote-like form in the cytoplasm. Videolaryngoscopy showed granulomatous and infiltrative lesions in the hypopharynx and larynx, hyperemia, and swollen vocal folds. There were also infiltrative lesions on the epiglottis, pyriform sinuses, and false folds. Results of viral and fungal serological tests, direct mycological examination, borehole azimuthal acoustic reflection imaging (BAAR), chest tomography, and respiratory function tests were all negative. Treatment with meglumine antimoniate was started but discontinued on day 22 due to phlebitis and fever. The patient was discharged on regression of all lesions and was referred for outpatient follow-up, but he did not return. He returned in 2018 because of recurrence and worsening of his condition. He presented with right-side nasal collapse and an erythematous lesion, which extended from the filter to the interior of the nasal orifice (Figure 2). Additionally, he had sinusitis with a foul-smelling discharge and blood clots. The sinus tomography showed nasal septum perforation and mucosal thickening of the maxillary sinus, with no other alterations on rhinoscopy. A 21-day treatment with intravenous amoxicillin/clavulanate was initiated.

**FIGURE 3:**
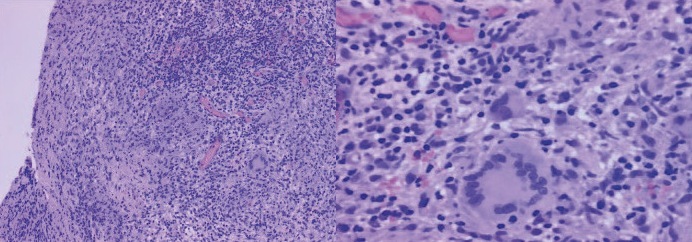
Histopathological examination results of the nasal mucosa, showing acute and chronic ulcerated inflammatory processes with giant cell reaction (Langerhans cells) and epithelioid histocytes, compatible with a diagnosis of leishmaniasis.

Serology for leishmaniasis was IgG-positive and IgM-negative and non-reactive for paracoccidioidomycosis. Nasal mucosa histopathology showed ulcerated squamous mucosa with seromucous glands in the chorion. There were marked acute and chronic inflammatory processes with a giant cell reaction (Langhans cells) and epithelioid histocytes, compatible with a diagnosis of leishmaniasis (Figure 3). Nasal fragment polymerase chain reaction (PCR) analysis using the primer set 120 base pair detected leishmaniasis. However, PCR examination did not allow for species evaluation. The patient was administered amphotericin B treatment for 14 days, after which his lesions improved, and he was discharged. He was re-evaluated three months later with continued healing and was followed-up by our outpatient service.

## DISCUSSION

MCL is a neglected public health issue. In the state of Rio de Janeiro, *L. (Viannia) braziliensis* has already been isolated. Between 2015 and 2017, 35 cases of MCL were confirmed^4^. Our patient lived in an impoverished neighborhood located in the lowlands of the Pedra Branca massif. This region is characterized by many residual areas of the Atlantic Forest, deforestation, and uncontrolled urbanization^3^.

Inhaling cocaine through the nose may destroy soft tissues and osteocartilaginous structures, progressing to the communications between the nasal, oral, and sinus cavities. These changes are probably due to the toxic effects of vasoconstriction, which can lead to ischemia. In our patient, we believe that substance abuse synergistically contributed to the observed deformities, buconasopharyngeal lesions, and disease worsening^5^. The role of cocaine in nasopharyngeal MCL recurrence is not yet fully understood. 

Cocaine inhalation causes local trauma. This case illustrates the progression of CL lesions after localized trauma. This trauma generates an inflammatory cascade that could favor Leishmania dissemination and lesion formation in previously infected patients. This hypothesis is supported by the local release of immunosuppressive cytokines and transforming growth factor-p, which exacerbates lesion development^6^.

Cocaine use increases monoamine levels, and dopamine helps regulate immunity. Compared with non-addicts, stimulant-addicted individuals showed significantly higher levels of CD4-cells, significantly lower levels of NK-cells, and no difference in CD8 and B-cell levels. Immunocompromise increases susceptibility to and accelerates progression of infectious diseases^7^. Therefore, trauma and immune changes mediated by cocaine may have contributed to the aggressive presentation and relapse of the disease.

In MCL, as the parasite is harbored within macrophages or Langerhans cells, the host must develop an effective cellular immune response. However, when it comes to mucosal leishmaniasis, there is a cellular overreaction associated with parasitic scarcity (hyperergic-pauciparasitic reaction) and a lack of adequate modulation of proinflammatory factors. Although this immunological reaction is capable of containing the multiplication of parasites, it is incapable of controlling the disease. This reaction is also responsible for tissue destruction proximal to Leishmania antigen particles^8^. It should be noted that the mucosal disorder does not follow this tendency in immunocompromised patients. This generally leads to an increased number of amastigotes^9^. The nasopharyngeal histopathology of our case shows this hyperergic-pauciparasitic pattern, with intense inflammatory infiltrate and only one macrophage with amastigote forms inside. Unexpectedly, we did not observe mucosal vasculitis secondary to cocaine use in our patient^5^. Complementary tests were also performed to exclude possible immunodeficiencies and-aside from cocaine-there was no history of use of other potentially immunosuppressive drugs.

MCL healing is rarely sterile, and the parasitic DNA is detected in wound scars years after clinical healing with appropriate treatment in most patients. Leishmania has been cultured from scar fragments, showing that this organism may be viable even years after it has been inside macrophages^10^. Initially, it was difficult to determine if the MCL recurrence was actually sinusitis associated with chronic cocaine abuse because the polymerase chain reaction (PCR) tests and serology were unreliable at the time of the first hospitalization.

Recurrence was diagnosed by knowing the natural history of mucosal leishmaniasis, which tends to be more recurrent and resistant to the characteristic clinical rhinoscopy and histopathologic nasal mucosa treatments^2^. Additionally, the patient’s good clinical response to liposomal amphotericin therapy assisted in making an appropriate diagnosis.

Currently, the drug of choice for MCL is meglumine antimoniate^4^. This was prescribed for 30 days during the first hospitalization in 2014; however, due to medication intolerance, the treatment was discontinued after 22 days. Because of this, the patient never showed complete healing, defined as the regression of all signs and symptoms and confirmed by an otorhinolaryngological examination up to six months after the conclusion of treatment^3^.

Amphotericin B is a polyene antibiotic with excellent *in vitro* activity in terms of destruction of intracellular and extracellular Leishmania^4^. In this case, amphotericin B was chosen because of the patient's history of adverse reaction to meglumine antimoniate.

By associating epidemiology, a condition that started in adulthood, with evolution and histopathology, we verified that the species involved in the condition was *L. (V.) braziliensis*, the main etiological agent involved in mucosal lesions^4^. Although Montenegro's intradermoreaction was not performed, it could have been used to differentiate the finding from the main anergic form, which is DCL^4^. However, *Leishmania (Leishmania) amazonensis*, the main etiological agent of DCL in Brazil, appears to a histopathologist as a large number of parasites inside vacuoles in macrophages and can present as tissue necrosis^10^. Also, our patient was not within the expected age range and did not exhibit the lesion polymorphisms that characterize DCL. 

In a study with 86 cases^11^, the main differential diagnoses of MCL were granulomatous diseases, especially paracoccidioidomycosis and leprosy, and basal or squamous cell carcinoma of the face. In this case, investigation of the differential diagnoses of necrotizing lesions in the mid-face was important because of the patient’s chronic use of inhaled cocaine and the trauma caused by it^12^. Further, cocaine inhalation may have triggered recurrence by aggravating the mucosal and nasopharyngeal leishmaniasis. Per journal guidelines, the authors adopted practices the procedures followed were in accordance with the ethical standards.
